# Reviewers’ Identity Cues in Online Product Reviews and Consumers’ Purchase Intention

**DOI:** 10.3389/fpsyg.2021.784173

**Published:** 2022-01-25

**Authors:** Ji Li, Xv Liang

**Affiliations:** Department of Marketing, Business School, Central University of Finance and Economics, Beijing, China

**Keywords:** online product reviews, identity cues, social support, review credibility, purchase intention

## Abstract

This research performs three experiments to investigate the influence mechanisms of identity cues in product reviews on consumers’ purchase intention, and to examine the effects of reference groups. The results indicate that: (1) identity cues in positive reviews have a significant positive impact on consumers’ purchase intention, while identity cues in negative reviews have a significant negative impact on consumers’ purchase intention; in addition, identity cues play a greater role in amplifying the impact of negative reviews on purchase intention; (2) emotional social support has a mediating role in the relationship between reviewers’ identity cues and purchase intention, while informational social support and review credibility only play significant mediating roles under all positive reviews scenario; and (3) identity cues of dissociative groups have a negative impact on purchase intention, whereas identity cues of in-groups or aspirational groups have a positive impact on the purchase intention. These findings complement existing research on online reviews and offer insights into the management and strategic oversight of product reviews for e-commerce platforms and merchants.

## Introduction

The product reviews are one of the important information sources for consumers to make their purchasing decision (Lee and Shin, 2014). Previous study has shown that the disclosure of review providers’ characteristics, such as personal identity, expertise, and reputation, can increase the perceived usefulness of online product reviews (Liu and Park, 2015). Reviewer disclosure of identity-descriptive information used by consumers to supplement or replace product information, is an important factor of consumers’ judgment on review helpfulness. Analyzing the secondhand data of Amazon book community, Forman et al. (2008) found that reviewer disclosure of identity information is one of the positive factors in subsequent sales prediction. In practice, enterprises generally assume that online reviewers with ambiguous identities will reduce consumers’ trust in the reviews they produce, negatively affecting the persuasive quality of product reviews. However, reviews with ambiguous identity information are not always less persuasive than those with clear identity information. Based on the concept of egocentric anchoring, Naylor et al. (2011) found that when consumers lack relevant information, they will automatically “filling in” ambiguous reviewers’ identities information blanks with their own information, which leads to consumers being more persuaded by reviews written by ambiguous reviewers than by those from dissimilar reviewers.

The disclosure of identity information in the above research is all straightforward and specific. Reviewers naturally are reluctant to provide their identity information for the privacy concerns. Except for the direct presentation of identity information, there are also such kind of words or phrases that does not directly indicate, but from which a reviewer’s identity can be indirectly inferred in reviews, making the reviewers’ image more vivid. For example, one online shopping review of chocolate says, “sweetness is moderate, my daughter likes to eat.” This is not just a simple introduction to product taste. Through the statement “my daughter,” this reviewer’s identity can be inferred; that is, the reviewer is a mother or father. We define such words as identity cues in product reviews.

Unlike identity information, these cues include subjective inferences regarding identities that are inferred from reviews. In fact, it is more common for reviewers to voluntarily disclose personal information by identity cues when reviewing personal experiences with products or services, which provides a more realistic reading experience for consumers. A total of 65,740 reviews on 36 products, including food, fitness, electronic products, books, accessories, daily necessities, and other categories, were obtained using an online crawler from the JD.COM website (a top online retailer in China). Among them, 15,122 reviews shared identity cues (mainly occupation and family information, such as being a father), accounting for about 23% of the total. The actual data indicate that consumers will express their identity cues (which can be used to infer reviewers’ age, occupation, family, and other personal information) in online product reviews.

When making online purchase decisions, consumers will intentionally refer to previous buyers’ product reviews. Consumers also tend to seek opinions from different types of people when buying different products. For example, when buying computers, consumers are more likely to consider the purchase choice of similar people (such as with a similar consumption level and similar personality). When buying necklaces, consumers are more likely to refer to the choices of those at a higher level (Li and Li, 2020). Furthermore, for the long-distance communication in virtual space, the recognition of identity cues may become an important basis for consumers’ establishment and maintenance of social connections, which makes people feel like communicating with “real” people. Therefore, we infer that the identity cues in reviews have similar influence on consumers’ purchase intentions as the reviewer disclosure of identity information.

This is what we will explore in the present research. Specifically, we will examine the effect of identity cues in product reviews on purchase intention, how it happens, and whether they have different influence regarding for different reference groups. It complements existing research on online reviews and offer valuable insights into the management and strategic oversight of product reviews for e-commerce platforms and merchants to increase sales.

## Research Hypotheses

### Reviewers’ Identity Cues and Purchase Intention

Product reviews include both buyers’ feedback and suggestions for potential consumers. Consumers share their thoughts and review other interactions in the comment area, thus being considered a form of online social community. Reviews provide consumers with product information. In addition to sharing a review regarding opinions and products or services experiences, reviewers’ identity cues convey information about the person who is transmitting the review (Brown et al., 2007). The previous research review shows that the direct disclosure of personal information has a positive impact on consumers’ purchase intention. Identity cues are another form of information disclosure, so it is reasonable to speculate that the impact of identity cues on consumers’ purchase intention is similar to that of direct disclosure.

In online shopping, all positive reviews and partial negative reviews (both positive and negative comments in the comment area) are the two most common situations. In circumstances in which reviews convey two types of valences (positive vs. negative), people pay more attention to negative ones. Previous literature has shown that negative reviews are more salient than positive reviews (Chen et al., 2011) and have a greater impact on consumers than positive ones (Skowronski and Carlston, 1989; Chevalier and Mayzlin, 2006; Chakravarty et al., 2010; Lee and Koo, 2012). Consumers believe that the expression of negative emotions in reviews is more persuasive (Li et al., 2011), and the description of product conditions is more accurate, which can more effectively help them reduce purchase risks in online shopping (Ahluwalia et al., 2000).

Based on the above, we infer that identity cues in positive reviews will increase the positive effect of reviews on consumers’ purchase intention. In contrast, identity cues in negative reviews can amplify the negative impact of the reviews on purchase intention. Further, the promoting effect of identity cues in positive reviews is weaker than that of negative reviews considering the dissymmetry influence of them. So we propose this hypothesis.

H1: Identity cues amplify the impact of product reviews on consumers’ purchase intention. In particular, compared with no identity cue, identity cues in positive reviews increase consumers’ purchase intention, whereas identity cues in negative reviews decrease purchase intention.

#### Mediating Role of Review Credibility

Review credibility is an influential aspect of reviews’ influence. Reviewers’ identity information, authority, and trustworthiness affect online review credibility (Park and Nicolau, 2015). Identity cues in reviews can result in consumers associating reviewers’ image and make the reviews perceived as credible information sources. This study assumes that personal reviews within identity cues help readers gain a more authentic experience of communication, and improve reviews credibility. Product reviews with high credibility are more persuasive (Song and Zhao, 2015) and promote consumers’ purchase intention. So we propose this hypothesis.

H2: Review credibility has a mediating role in the relationship between identity cues and purchase intention.

#### Mediating Role of Social Support

Social support is usually defined as help offered by someone with whom the recipient has an interpersonal relationship, including family members, friends, colleagues, and significant others (Zimet et al., 1988). These relationships are based on social interactions and can be virtual, implied, imagined, real, momentary, or ongoing (Luo and Chen, 2018). Social support also exists in the online virtual environment (Liang and Wei, 2008), and online channels have become an increasingly important means for people to receive and deliver social support (Qian et al., 2020). Social support has three dimensions, including tangible, informational, and emotional support (Schaefer et al., 1981). Among them, informational support refers to information shared to help solve problems, often in the form of suggestions, recommendations, and other forms of useful advice. Emotional support is the provision of emotional care, such as encouragement. Due to the virtuality of cyberspace, online social support is rarely presented in the form of tangible support, but more informational and emotional support (Coulson, 2005). Social support obtained through the internet can reduce loneliness, depression, stress, and other adverse psychological reactions (Valkenburg and Peter, 2007; Yao et al., 2018), and promote physical and mental health. Social support can relieve life pressure and improve personal happiness. The experience of positive emotions can positively influence consumers’ purchase intention (Yan and Wang, 2017).

Identity cue is the premise of shaping the sense of social connection and perceiving social support in the virtual environment. It makes people feel like communicating with “real people” in virtual online shopping, and plays a role in enhancing perception. On the one hand, identity cues help consumers obtain emotional positive support from reviewers by reading reviews. On the other hand, the existence of identity cues shortens the subjective distance between consumers and reviews, so that consumers are more likely to recognize and perceive the reviewers’ informational support about the product itself and problem-solving methods. Therefore, we assume that the sense of social support gained by reading such reviews will increase purchase intention. In this study, social support (emotional and informational support) is assumed to play a mediating role between reviewers’ identity cues and consumers’ purchase intention.

H3: Social support (emotional and informational support) has a mediating role in the relationship between identity cues and purchase intention.

### Influence of Reference Groups

Reference group refers to individuals or groups that are considered for reference and comparison when individuals make purchase decisions (Jiang et al., 2009). According to the degree of social identification, reference groups can be divided into in-groups and out-groups, while out-groups include aspirational reference groups and dissociative reference groups (Liu et al., 2017). Previous studies have found that consumers’ choice of products and brands is influenced by these reference groups. Consumers prefer products that are endorsed by an aspirational group (Whittler and Spira, 2002). When brand image is consistent with an in-group, consumers’ self-brand connection can be enhanced (Escalas and Bettman, 2003). In contrast, consumers will show negative evaluation and decision-making behavior toward products endorsed by dissociative groups (Berger and Heath, 2008).

The influence of reference groups’ value evaluation refers to consumers’ desire to establish a psychological connection with a positively perceived reference group. Identities form a basis for social classification (Aquino and Reed, 2002). When shopping online, the identity cues can allow consumers to identify the reviewer’ social information, so as to enrich the image of reviewers and infer their group. Through this process, consumers can connect emotionally and psychologically with aspirational groups to improve their self-image (Alvarez and Fournier, 2016), establish connections with in-groups to enhance the sense of group attachment (Escalas and Bettman, 2003), and exclude dissociative groups to avert their purchasing choices (Berger and Heath, 2008). The following hypotheses are therefore proposed.

H4a: Reviews containing identity cues from in-groups and aspirational groups positively affect purchase intention.

H4b: Reviews containing identity cues from dissociative groups negatively affect purchase intention.

## Materials and Methods

Three studies were conducted to test these hypotheses. Study 1 examined the impact of identity cues in reviews on purchase intention, and the mediating roles of social support and review credibility. Study 2 further examined the difference of identity cues’ amplification between positive and negative reviews on purchase intention. Study 3 examined the difference in the impact on purchase intention of reviews containing identity cues from in-groups, aspirational groups and dissociative groups.

### Methods

The experiments are designed as a shopping task for the participants, in which they will read some product reviews and then indicate their purchase intention. Before the experiments, it is necessary to determine the total number of reviews presented to the participants and the number of negative reviews among them. So we conducted the following two pilots.

#### Pilot 1

We did a 50-person test that 88% of the participants (48% male, averagely aged 27.7, SD = 6.8, 28% students, 50% staff, 8% farmer and factory-hands) would consult product reviews after browsing product information. By further observing the online shopping process recorded by participants^1^, we found that 68% of consumers who shopped on the computer interface were likely to read 30 reviews (comment interface is page turning, and per page has 10 reviews), and consumers who shopped on the mobile interface read 21.2 reviews (comment interface is sliding) averagely. Because our experiments are carried out on the computer, so we decided to use 30 reviews in the experiment.

#### Pilot 2

In order to determine the number of negative reviews, we conducted a 30-min experiment with 50 college students (50% male, averagely aged 19.8, SD = 0.90) in the university. Participants were asked to search the designated five products (notebook, folder, chocolate, umbrella, book bag) on the computer, and are promised that they would be able to buy one of their favorite products among the five products at the expense of this experiment funds. Their search process was recorded by computer screen. Among the total 235 searches, 233 searches opened the product review interface for browsing and page turning (averagely page turning times 3.5, SD = 1.82). We manually coded the participants’ review browsing behavior. In 19.3% of the search behavior, when the participants saw 1 negative review, they ended the comment reading, and then exited the product page. 19.7% of the search behavior, when participants see 2 negative reviews, they exit this product page. 30.9% of the search behavior, when participants see 3 negative reviews, they exit the product page. In 12.4% of the search behavior, when participants see 4 or more negative reviews, they exit the product page. 17.6% of the search behavior did not have negative reviews. According to the results, we decide that 3 negative reviews are enough under partial negative reviews scenario.

### Materials

To avoid the impact of the particularity of product’s target group on the type of identity cues (such as the applicability of clothing products to men or women, or the matching degree of health care products to age) and the preference of different groups for product selection, all the products selected in the experiments are very common and applicable to a wide range of individuals (e.g., electric toothbrush, Chinese wolfberry, portable juicer, albumen powder, water cup, neck massager, backpack, chocolate, toothpaste, coffee)^2^. The products of each experiment are randomly selected from this product collection. As the research object is online shopping reviews, this study selects the real products’ reviews on JD.COM as the experimental material source. Using the portable juicer as an example, Table 1 presents the specific reviews^3^.

**TABLE 1:** Review presentation.

Review type	Presence of identity cues	Review on the content
Positive reviews	Yes	158013qhi 2021-2-26 09:05 Too convenient. I had two cups of milk (pitaya, and orange). I can take it **on business** in the afternoon.
		Melon 2020-12-30 16:59 I really like it. It’s better than expected, and also convenient for **my children** to live in **school**.
Negative reviews	Yes	290333qhi 2021-4-26 01:05 I was going to buy it for use **in school**. Today I tried it, but it was bad! It can’t be repaired at all!
		Ha*** love watermelon 2021-2-30 11:20 Don’t buy it. If you buy it, you’ll be fooled. It’s useless. I’m preparing to return it downstairs. **My boss** said that fortunately she didn’t buy it.
Positive reviews	No	182kue***2 2021-02-11 12:31 It’s very small and cute. Do not take up space. Beautiful, suitable for summer.
		156437vil 2020-12-16 09:54 Very good, and very convenient! Successfully replace the heavy juice machine! It’s easy to clean.
Negative reviews	No	I****VE 2021-1-26 09:12 I bought it to make a milkshake. It broke after one use. And it’s really too small to use.
		15y22e 2021-01-13 16:34 There is a smell of plastic. I dare not use it. I washed it with hot water several times, but it still smells.

*The bold terms mean cue words reflecting identity information.*

## Study 1

### Participants and Procedure

Study 1 examined the impact of identity cues in reviews on purchase intention, and the mediating roles of social support and review credibility. 416 Participants (52.2% male, averagely aged 27.6, SD = 9.0, 16.6% student, 55.5% staff, 12.0% factory-hands) in China participated in this experiment from website, using Credamo survey platform. Participants were randomly assigned to one condition in a 2 (scenarios: all positive reviews, partial negative reviews) × 2 (identity cues: with, without) between-subjects design. We selected Chinese wolfberry and water cup as the products by randomly selecting from the public product collection.

Each participant was randomly matched with a situation (e.g., Chinese wolfberry, and identity cues under partial negative reviews scenario). The experiment was carried out with the following three steps. First, participants read the product information (including product picture, name, price, function description), then conducted a purchase intention survey and asked them if they are willing to read the follow-up product reviews. If participant is unwilling to read product reviews, the experiment will be ended. 97% of the participants chose to read product reviews. The 30 product reviews were presented in the form of turning pages (each page has 10 reviews). After reading reviews, participants were asked to answer questions that are not related to the product reviews, such as “do you remember the product’s color?” “how many products are presented in the picture?” and so on. Finally, after answering these irrelevant questions, participants were measured on their product purchase intention (7-point Likert scale, 1 = “strongly disagree” and 7 = “strongly agree”), social support and review credibility.

### Measures

Regarding the measurement of online social support, this study divided online social support into two categories of emotional and informational social support by comprehensively referring to the relevant studies of Rosenbaum (2006) and Zhu et al. (2016), with corresponding modifications according to the characteristics of online reviews. To measure review credibility, a scale designed by Cheung et al. (2009), including three items, was adopted. All measurements used a 5-level Likert scale, as shown in Table 2.

**TABLE 2:** Measurement variables.

Measurement variables	Item
Emotional social support	In the reviews, I felt supported. In the reviews, I felt comforted and encouraged. In the reviews, I could get a sense of belonging.
Informational social support	In the reviews, someone gave me advice on buying products. In the reviews, someone gave me information about buying products. In the reviews, someone helped me find the product problems, and put forward relevant suggestions.
Review credibility	I believe the reviewers are honest. I believe the reviewers are true. I believe the review information is reliable.

As indicated in Table 3, Cronbach’s alpha of all variables is greater than 0.8, indicating that the scale exhibited high internal consistency and strong reliability.

**TABLE 3:** Reliability analysis results.

Variable	Item number	Cronbach’s alpha (α)
Emotional social support	3	0.818
Informational social support	3	0.832
Review credibility	3	0.907

To further verify the validity of measurements, we performed the confirmatory factor analysis. The goodness of fit measurement model’s index included a ratio of chi-square to freedom of 3.001 < 5; root mean square of approximate error of 0.062 < 0.08; goodness of fit index of 0.936 > 0.90; comparison fitting index of 0.976 > 0.90; norm fitting index of 0.952 > 0.90; saving norm fitting index (PNFI) of 0.797 > 0.50; and saving goodness of fit index (PGFI) of 0.665 > 0.50. All of the above fitting indexes meet the requirements of the fitting standards, indicating a good fitting effect of the measurement model. Average variance extraction (AVE) and combined reliability (CR) were calculated by measuring the standardized factor loads of each observation index in the model, obtaining an emotional support AVE of 0.732 and CR of 0.897; informational support AVE of 0.623 and CR of 0.829; and review credibility AVE of 0.749 and CR of 0.947. As the average extraction variation of each latent variable is greater than 0.5, and the CR is greater than 0.7, this further indicates that the scale has good internal consistency and good structural validity.

### Results and Discussion

**FIGURE 1:**
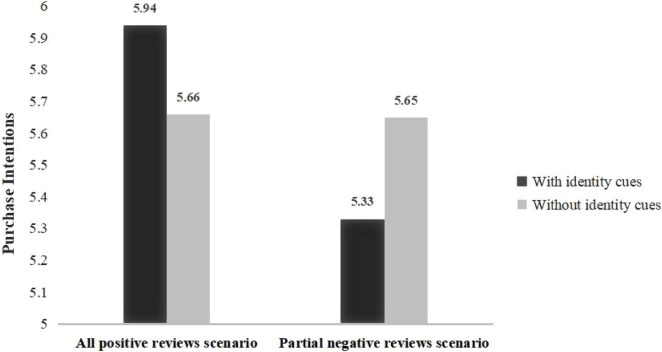
Comparison of purchase intentions after reading product reviews.

The results of variance analysis include three findings. First, before reading the reviews, the samples in the four groups show no significant difference in purchase intention [*M*_*all positive reviews with identity cues*_ = 5.57, SD = 1.26; *M*_*all positive reviews without identity cues*_ = 5.63, SD = 1.03; *M*_*partial negative reviews with identity cues*_ = 5.63, SD = 1.02; *M*_*partial negative reviews without identity cues*_ = 5.56, SD = 1.04; *F* (3, 412) = 0.131, P = 0.951]. Second, when all reviews are positive, purchase intention with identity cues in reviews is higher than those without identity cues [*M*_*with identity cues*_ = 5.94, SD = 1.04; *M*_*without identity cues*_ = 5.66, SD = 0.85; *F* (1, 206) = 4.457, *P* < 0.05], indicating that the main effect of identity cues on purchase intention is significant. Third, purchase intention with identity cues in reviews is lower than those without identity cues under partial negative reviews scenario [*M*_*with identity cues*_ = 5.33, SD = 1.22; *M*_*without identity cues*_ = 5.65, SD = 1.12; *F* (1, 206) = 15.421, *P* < 0.05] (see Figure 1). Although there are only three negative reviews per product (total reviews’ number is 30), this result shows that identity cues seriously amplify the negative impact of negative reviews on purchase intention. Therefore, we inferred that the effect of positive and negative reviews on purchase intention amplified by identity cues is different. This research specifically explores this phenomenon in Study 2.

The results of the bootstrap mediating effect test (sample size 5,000, 95% confidence interval) under all positive reviews scenario demonstrate that a significant direct effect of identity cues on purchase intention (LLCI = 0.0069, ULCI = 0.4579, *t* = 2.032, *P* < 0.05). The mediating effects (see Table 4) of emotional social support (LLCI = 0.0524, ULCI = 0.4475), informational social support (LLCI = 0.0481, ULCI = 0.3432), and review credibility (LLCI = 0.0795, ULCI = 0.4487) are significant. In addition, the regression results show that the identity cues in reviews have a positive impact on emotional social support (β = 0.6250, *P* = 0.0276), information social support (β = 0.7154, *P* = 0.0071) and review credibility (β = 0.8269, *P* = 0.0028), while emotional support (β = 0.3581, *P* < 0.001), informational support (β = 0.2732, *P* < 0.001) and review credibility (β = 0.3699, *P* < 0.001) positively affect purchase intention. In sum, the indirect effect consists of two pathways: identity cues → social support → purchase intention, and identity cues → review credibility → purchase intention.

**TABLE 4:** Mediating effects between identity cues and purchase intention under all positive reviews scenario.

Mediation path	Effect value	LLCI	ULCI	SE
Identity cues → Emotional social support → Purchase intention	0.24	0.0524	0.4475	0.101*
Identity cues → Informational social support → Purchase intention	0.17	0.0481	0.3432	0.074*
Identity cues → Review credibility → Purchase intention	0.22	0.0795	0.4487	0.094*

*p < 0.05.

We test the mediation mechanism under partial negative reviews scenario (sample size 5,000, 95% confidence interval). The results (see Table 5) show that the mediating effect of emotional social support (LLCI = −0.4182, ULCI = −0.0197) is significant. The mediating effects of informational social support (LLCI = −0.0509, ULCI = 0.1237) and review credibility (LLCI = −0.1043, ULCI = 0.0610) are not significant. The regression results show that the identity cues in negative reviews (β = −0.6859, *P* = 0.0348) have a negative impact on emotional social support, while emotional support (β = 0.3737, *P* < 0.001) positively affects purchase intention. The impact of identity cues and informational social support is not significant (β = 0.1057, *P* = 0.53), but informational support (β = 0.2562, *P* < 0.001) positively affects purchase intention. Identity cue (β = 0.9782, *P* < 0.001) has a positive impact on review credibility, but the impact of review credibility and purchase intention is not significant (β = −0.0383, *p* = 0.361).

**TABLE 5:** Mediating effects between identity cues and purchase intention under partial negative reviews scenario.

Mediation path	Effect value	LLCI	ULCI	SE
Identity cues → Emotional social support → Purchase intention	−0.22	−0.4128	−0.0197	0.102**
Identity cues → Informational social support → Purchase intention	0.03	−0.0509	0.1237	0.044
Identity cues → Review credibility → Purchase intention	−0.02	−0.1043	0.0610	0.041

*p < 0.05 and **p < 0.01.

Compared with no identity cues, consumers’ purchase intention is significantly decreased after browsing reviews with identity cues under partial negative reviews scenario. In this process, consumers are attracted by negative reviews. The existence of identity cues makes them feel like “having a real person” make complaints about this product, which lead to the decrease of emotional social support. So emotional social support plays a negative mediating role in the relationship between identity cues and purchase intention. At the same time, negative reviews are more about bad product experience but not how to solve the problems. Therefore, identity cues in negative reviews have no influence on informational social support. Although identity cues increase the credibility of negative reviews, we think this promoting effect may not be enough to affect the purchase intention because consumers are more likely to trust negative reviews no matter with or without identity cues.

The results of Study 1 demonstrate that identity cues significantly amplify the impact of product reviews on consumers’ purchase intention, confirming Hypotheses 1. Hypothesis 2 and 3 are verified that social support and review credibility implying a significant mediating effect on this relationship under all positive review scenario. In the situation of partial negative reviews, only emotional social support plays a significant mediating role.

## Study 2

### Participants and Procedure

Study 2 was designed to further examine the difference of identity cues’ amplification between positive and negative reviews on purchase intention under partial negative reviews scenario. 292 Participants (52.1% male, averagely aged 29.1, SD = 8.8, 21.6% student, 51.7% staff, 12.3% factory-hands) in China participated in this experiment from website, using Credamo survey platform. The study examined four experimental groups (identity cues which appeared only in positive reviews vs. appeared only in negative reviews vs. no identity cue vs. both positive and negative reviews have identity cues). We selected juicer and toothpaste as the products, and performed the same procedure as study 1.

### Results and Discussion

**FIGURE 2:**
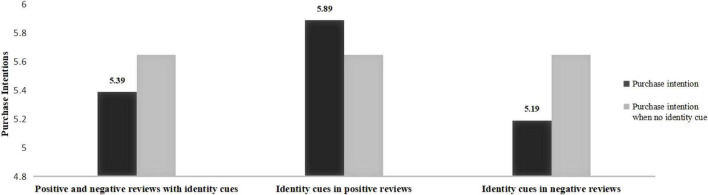
Comparison of purchase intentions after reading product reviews.

A variance analysis of purchase intention before and after reading the reviews was conducted. First, before reading the reviews, the samples in the four scenarios have no significant differences [*M*_*positive reviews with identity cues*_ = 5.55, SD = 0.88; *M*_*identity cues in negative reviews*_ = 5.60, SD = 0.80; *M*_*no identity cue*_ = 5.51, SD = 0.73; *M*_*positive and negative reviews with identity cues*_ = 5.61, SD = 1.03; *F* (3, 288) = 0.204, *P* = 0.893] in purchase intention. Second, samples in the group which identity cues only appeared in positive or negative reviews have significant differences in purchase intentions after reading reviews (see Figure 2). Compared with no identity cue in product reviews, (1) consumers’ purchase intention is higher when identity cues only appeared in positive reviews [*M*_*no identity cue*_ = 5.65, SD = 0.89; *M*_*identity cues in positive reviews*_ = 5.89, SD = 0.79; *F* (1,144) = 3.025, *P* < 0.1]. (2) Consumers’ purchase intention is lower when identity cues only appeared in negative reviews [*M*_*no identity cue*_ = 5.65, SD = 0.89; *M*_*identity cues in negative reviews*_ = 5.19, SD = 1.10; *F* (1,150) = 7.999, *P* < 0.01]. Third, compared with no identity cues in product reviews, when identity cues appear in both positive and negative reviews, the purchase intention is lower [*M*_*no identity cue*_ = 5.65, SD = 0.89; *M*_*positive and negative reviews with identity cues*_ = 5.39, SD = 0.96; *F* (1,152) = 2.998, *P* < 0.1].

The results of Study 2 demonstrate that reviewers’ identity cues have a significant impact on consumers’ purchase intention. Specifically, identity cues in positive reviews positively influence consumers’ purchase intention, and identity cues in negative reviews have a negative impact on purchase intention, confirming Hypotheses 1 again. In addition, the results of purchase intention after reading positive reviews (or negative reviews) with identity cues and reviews without identity cues show that, compared with positive reviews, identity cues play a greater role in amplifying the impact of negative reviews on purchase intention. This conclusion is also proven by the difference of purchase intention between the two groups of “no identity cues in reviews” and “identity cues in both positive and negative reviews,” from the side.

## Study 3

### Participants and Procedure

Study 3 examined the influence of reviewers’ identity cues from in-groups, aspirational groups, and dissociative groups on consumers’ purchase intention. The experiment was conducted using (reference groups: in-groups, aspirational groups, and dissociative groups vs. no identity cue) a single factor design. Neck massage machine, coffee, and neutral perfume are selected as experiment products randomly. All of 30 reviews for each product were positive.

Because the participants are undergraduates, in-group’s identity cues include dormitories, roommates, libraries and classrooms. Aspirational group’s identity cues include white-collar workers, clubs, mental work occupations, high-income and high-end communities. Dissociative groups cues include elderly people, low-income, poor living environment, etc.^4^ 495 college students (43.6% male, averagely aged 20.0, SD = 1.55) in China participated in this experiment. Each participant was randomly matched with a situation (e.g., coffee, reviewers’ identity cues from in-groups), and the experimental procedure was the same as that of study 2.

### Results and Discussion

**FIGURE 3:**
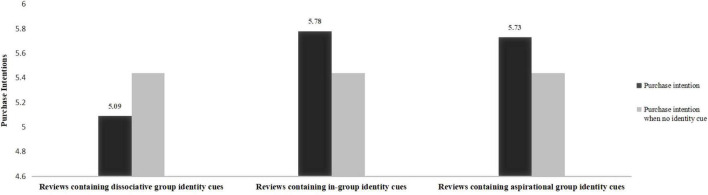
Comparison of purchase intentions after reading product reviews.

Before reading reviews, there is no significant [*F* (3, 491) = 1.071, *P* = 0.361] difference in purchase intention between the samples in the four scenarios of reading reviews containing identity cues from in-groups, aspirational groups, dissociative groups and the reviews without identity cues. After reading reviews, the samples with or without reviewers’ identity cues in the reviews reveal a significant difference in consumers’ purchase intention [*F* (3, 491) = 10.186, *P* < 0.001]. Specific analysis results show that compared with no identity cue in product reviews, (1) consumers’ purchase intention is higher when reviews contained in-group and aspirational group identity cues [*M*_*reviews containing in*–*group identity cues*_ = 5.78, SD = 1.07; *M*_*reviews without identity cues*_ = 5.44, SD = 0.95; *F* (1, 242) = 0.6653, *P* < 0.05; *M*_*reviews containing aspirational group identity cues*_ = 5.73, SD = 0.99; *M*_*reviews without identity cues*_ = 5.44, SD = 0.95; F (1, 243) = 5.133, *P* < 0.05]. (2) Purchase intention is lower when reviews contain identity cues from dissociative groups [*M*_*reviews containing* dissociative group identity cues_ = 5.09, SD = 1.37; *M*_*reviews without identity cues*_ = 5.44, SD = 0.95; *F* (1, 238) = 5.403, *P* < 0.05] (see Figure 3). The results indicate that reviews containing identity cues from in-groups and aspirational groups have a positive impact on purchase intention, and those containing identity cues from dissociative groups will negatively affect purchase intention. In addition, the ANOVA analysis for in-groups and aspirational groups indicates that identity cues from in-groups and aspirational groups have no significant difference on purchase intention [*M*_*reviews containing in*–*group identity cues*_ = 5.78, SD = 1.07; *M*_*reviews containing aspirational* group identity cues_ = 5.73, SD = 0.99; *F* (1, 253) = 0.168, *P* = 0.682].

Experimental results indicate that the influence of identity cues on consumers’ purchase intention was moderated by reference group. Compared with reading reviews without identity cues, the reviews containing identity cues from dissociative groups will negatively influence purchase intention, identity cues from in-groups and aspirational groups have a positive impact on consumers’ purchase intention, confirming Hypotheses 4.

## Discussion and Conclusion

This study verifies that reviewers’ identity cues amplify the impact of product reviews on consumers’ purchase intention, and the extent to which voluntary identity cues have a role in positive and negative reviews. The influence of the amplification of negative reviews by identity cues on purchase intention is greater than that of positive reviews, resulting in insignificant, or even negative influence of identity cues on purchase intention under partial negative reviews scenario. This study examines the mediating roles of emotional social support and review credibility. For consumers who have read the product information itself, they hope to confirm their purchase or non-purchase decision through “real person” reviews (credible) and get a sense of support. Reviews containing reviewers’ identity cues (vs. those without identity cues) can increase readers’ perceptions of social support and review credibility, influencing their purchase intention. This study also verify the influence of reference group. As for identity cues from different reference groups in the reviews, significant differences of influence on consumers’ purchase intention were found. In comparison to reviews without identity cues, those containing identity cues from dissociative groups have a negative impact on purchase intention, whereas identity cues from in-groups and aspirational groups have a positive impact on purchase intention.

### Research and Managerial Implications

This research makes several theoretical and practical contributions. Theoretically, we increase the identity information disclosure literature of product reviews in online shopping. We expand the form of information disclosure in product reviews and examine how identity cues (voluntary and indirect) in product online reviews affect consumers’ purchase intention, rather than direct identity information display. Specifically, previous studies have confirmed that reviewers’ direct identity information disclosure will significantly affect review credibility, and has been applied in the marketing. However, few existing studies further distinguish the different forms of identity disclosure. Identity cue is an indirect and vague form of identity information disclosure, and is more common in real online shopping reviews. This study verifies the impact of voluntary implicit identity cues on consumers’ purchase intention, and discusses the multiple boundary conditions of the main effect. This study provides an interesting perspective for the field of online reviews. In addition, we also enrich the literature on social support in the Internet and the impact of reference groups on consumers’ purchase intention.

The conclusions offer important marketing implications for successfully leveraging the influence of product reviews on consumers’ purchase intentions in online shopping environments. First, e-commerce platforms and registered merchants should actively promote communication between the reviewers, potential consumers, and others. They should take measures to increase reviews and replies to improve the cohesion of the existing virtual community. Even by providing review templates, platforms can guide buyers to express themselves at a deeper level. Second, merchants should improve the ability to manage negative reviews and expediently address negative reviews. To avoid the magnifying effect of identity cues on negative reviews, reviews containing identity cues should be prioritized in cases of limited oversight resources. In addition, merchants can consider using labels to manage product reviews so that consumers are able to navigate to relevant reviews that are produced by in-groups and aspirational groups, and by pass product reviews of dissociative groups through ranking and appropriate placement.

### Limitations and Directions for Future Research

This study has some notable limitations suggesting further research. First, although we tried to minimize the demanding effect in the process of experiment, some participants may still have chance to get the purpose of the experiment since we measured the purchase intention twice before and after they read the product reviews. In order to make the results more valid, the experiment design could be improved in the future.

Second, individuals’ personality characteristics and cognitive process will influence the effect of reviewers’ identity cues on consumers’ purchase intention. Compared with secure attachment, people with an insecure attachment receive less social support and are less satisfied with the social support they receive (Liu and Ma, 2019). Individuals with differing regulatory focus could have different attitudes and decisions regarding the reviews of emotional strength (Zhang et al., 2018). In comparison to negative information, promotion-focused consumers have been shown to be more sensitive to positive information (Yu et al., 2016), whereas prevention-focused consumers are more sensitive to negative information (Tian et al., 2014). In addition, whether identity cues in positive reviews have a negative effect on purchase intention under influencing factors such as uniqueness seeking can also be considered.

Third, the impact of the review form can be considered. In general, the longer the review (Mudambi and Schuff, 2010), the more likely it is to include emotional expressions and identity cues. An excessive number (Park et al., 2007) of reviews will lead to the depletion of consumers’ psychological energy, and the depth of reviews will affect the perceived usefulness of reviews (Fu and Wang, 2015). How to properly control the length and number of reviews in the experiment is a challenging research.

Last, products can be divided into tangible and intangible products according to tangibility, as well as search products, experience products, and trust products according to consumers’ understanding of product characteristics. Compared with search products, the review content of experience products is mostly personal experience, and contains more identity cues. When purchasing experience products online, consumers will conduct more frequent and in-depth search behavior (the number of reading reviews increases), and are more vulnerable to others’ opinions (Lim et al., 2015). In the future, the regulatory effect of products can be investigated using different classification methods.

## Data Availability Statement

The raw data supporting the conclusions of this article will be made available by the authors. Further inquiries can be directed to the corresponding author.

## Ethics Statement

Ethical review and approval was not required for the study on human participants in accordance with the local legislation and institutional requirements. Written informed consent from the participants was not required to participate in this study in accordance with the national legislation and the institutional requirements.

## Author Contributions

JL and XL designed the study and wrote the protocol. XL was responsible for data collection, under the supervision of JL. XL conducted the statistical analyses and wrote the first draft of the manuscript. Both authors edited subsequent drafts and have approved the final manuscript.

## Conflict of Interest

The authors declare that the research was conducted in the absence of any commercial or financial relationships that could be construed as a potential conflict of interest.

## Publisher’s Note

All claims expressed in this article are solely those of the authors and do not necessarily represent those of their affiliated organizations, or those of the publisher, the editors and the reviewers. Any product that may be evaluated in this article, or claim that may be made by its manufacturer, is not guaranteed or endorsed by the publisher.

## Footnotes

1 Participants were asked to record the process of browsing products on computers and mobile phones, and upload them.

2 By viewing the product introduction and applicable groups description.

3 See Supplementary Appendix for more reviews.

4 We recruited 20 senior students (50% male, averagely aged 19.7, SD = 0.75) in the university to select reviews on the identity cues of reference groups (in-group, aspirational groups, and dissociative groups). The participants were randomly divided into three groups to classify and select the reviews at the same time. Each participant selected out the reviews with identity cues of different reference groups from 2,000 reviews on this three specified product. Finally, reviews with the same classification constitute the database of study 3. In the experiment, reviews will be randomly extracted from this database according to the previous reference group code.
