# Supplementary material for: Reviewers’ Identity Cues in Online Product Reviews and Consumers’ Purchase Intention
Source: Front Psychol. 2022 Jan 25;12:784173. doi: 10.3389/fpsyg.2021.784173 (PMC8822043; doi:10.3389/fpsyg.2021.784173)
Supplement: Supplementary file 1 [file Data_Sheet_1.pdf]

Study 1：

1. Water cup, all positive reviews, with identity clues

jd_ 133585fdi 2021-02-24 09:55
The **women's Day** gift bought for the **female employees** at the bottom. The quality of the health cup is very good and the workmanship is exquisite. My **girlfriend** should like it very much.

PafatvpjpHBA 2020-10-30 11:57
It's very good. The workmanship is good. The order was placed last night and arrived in the morning. It was given to the **staff** on Thanksgiving. The style is very beautiful and practical. There are many main functions. I bought 50 sets and several boxes are very heavy. I believe everyone will be satisfied with this health care gift.

jd_ 150416esd 2020-09-24 17:26
It's a very good one. The gifts given to **employees** in the store during the Mid Autumn Festival are much better than expected. It's very practical. I used them at home first. It's good. I can stew and boil them. The logistics is very fast. They arrive the next day. The workmanship is also good and exquisite

j***a 2020-11-11 09:19
This is a gift for the annual meeting prepared by the **company**. I took it home and opened it for use. It's really good. Many things can be stewed. For example, the lotus seed and red jujube soup I stewed is very nourishing. The size of the cup is also moderate, about 500ml, with two bowls. The whole health cup set looks very high-grade and I like it very much. Like friends can start.

jd189755svv 2020-09-22 11:32
The packaging is very good. They give Christmas gifts to **employee**s. It's a very good style. You can recommend this one.

srkKqJoRkNgD 2020-11-21 15:14
I purchased 100 sets for the **company**. The quality of the goods is very good. Everyone in the office sends one set. Mine is very practical in the office. It is very convenient to cook flower tea and occasionally stew porridge.

Ju * * * 2 2020-11-09 16:26
The health cup is a very good gift for **employee**s. The end of the year gifts and year-end prizes are given to **employee**s. Employees like it very much. The cup has a good sense of design, powerful hot cooking function and is very practical.

j***c 2020-12-08 11:28

The cup style is pretty good, and the heating effect of the coaster is very good. This time, I bought two or three hundred gifts for the **employee**s and **leader**s of each branch for the third anniversary. I'm generally very satisfied

j***s 2020-12-15 15:29
The company's annual meeting gifts that **my boss** asked me to buy are of good quality on the whole. It's also very convenient for me to cook. The staff liked it and the boss was very satisfied.

j***V 2020-11-30 14:58
The goods have been received. The gift for **my mother** is exquisite in packaging, small and portable, and the electricity consumption is not large. It does not violate the company's regulations. Drink it warm, do not drink it cold, do not overflow and do not paste the bottom. It's really great.

Xiaosu melon 2020-12-30 16:59
In terms of compactness, it doesn't cost electricity and has a high appearance value. It's very convenient to use it **at work in the company**. Logistics is also fast. This health cup is very good, the cooking is also very good, the appearance is good, and it is very good to use. Nice new year gift.

jd_ 1861665941 2021-01-11 15:19
It's cold. A health cup is really practical. This is the gift I bought for **my girlfriend**. It's really easy to use. It can cook a lot of things. It has an atmospheric appearance. It's green and inlaid with gold. It's very high-grade. I like it very much.

jd_ 182505ykue 2021-01-13 16:34
It's also very convenient to cook pearl milk tea with it. Personally, I think it's great. The amount of cooking is also enough for one person, which is very practical and convenient. **My girlfriend** is the most satisfied Christmas present.

jd_ 156437vil 2020-12-16 09:54
The annual meeting accompanying gift given to **employee**s has a gift box, the function is also very practical, and it is very convenient to cook. Cook corn ribs soup. It tastes good.

jd_ 387777230 2020-09-11 05:35
A gift for **teachers' day**. I like it very much.

BLACK * * * 6 2020-10-20 00:40
It's very practical to give gifts to customers and the **company**'s Mid Autumn Festival employees on the national day. It has many functions. It's convenient to stew. It's very convenient to go out and use inside. It's relatively good to give gifts. I've seen a lot of functions. I bought a dozen or so and plan to renew some

j***8 2020-5-26 08:55
The gift of 520 for my wife happened to be our **wedding anniversary**, so I bought this kind of

health preserving electric stew cup. The style is more exquisite and has more practical functions. On the whole, it's still very good.

claw 2020-12-26 10:51
In the **leader**'s office, the leader bought it, so he also bought one at home, which is easy to use.

jd_ Pig's 2020-10-22 07:10
Teacher's Day gift for teachers, good. I just lose without a gift bag.

pencil head 2021-01-24 13:53
Women's Day gifts. Every female colleague has holiday gifts for female employees. They are very convenient for cooking bird's nests. It feels very easy to use. A good gift.

super21jdmau 2020-09-13 17:01
The Christmas gift bought in advance for the female colleague in pursuit. The **female colleague** liked it very much. She said that the appearance was very good. She liked it very much. She often used it to make tea. I was very happy to see her use it.

Spuerman1222 2021-02-09 23:27
The **women's Day** gift I bought for my mother, the health cup, is very suitable. The quality is very good. I feel very good. My mother likes it very much.

8***3 2020-09-11 17:40
very nice. It's very practical and cost-effective. It's a birthday gift for your **girlfriend**. She can use it to cook porridge.

hhhhhhh1 2020-05-15 12:17
Super beautiful color, especially suitable for **girl**s, easy to use, economical and affordable.

wsqdm123 2020-09-18 17:46
Especially girls are very practical. I have one in **my office**. I often cook snacks and health tea. The quality is very good

Roseyo43 2020-12-26 10:52
It is very suitable for making gifts. This health cup is a holiday gift I intend to give to my **mother** before women's day. The workmanship of the health cup is very meticulous

jd_ 158013qhi 2020-12-26 09:05
The prizes purchased at the company's annual meeting are pretty good. There are a lot of **employee**s. This time they need to be in a hurry, and the logistics is very fast. It was delivered as expected. Thank you. The gift health cup is very good. I really like it.

j***1 2021-01-28 09:48
As a company gift, **colleague**s like it very much.

Luka_sky 2020-07-14 12:22
Style: Great
Applicable crowd: it's good to send t**eacher**s.

jd_ 180619ryh 2020-09-12 16:39
It was sent to the **leader**. The feedback was very good. Thank you for your considerate service.

---

2. Water cup, all positive reviews, no identity clues

jd_ 133585fdi 2021-02-24 09:55
The health cup is of very good quality and exquisite workmanship. I like it very much.

PafatvpjpHBA 2020-10-30 11:57
Very good. The workmanship is good. The order was placed last night and arrived in the morning. The style is very beautiful and practical. There are many main functions. I bought 50 sets and several boxes are very heavy. I believe everyone will be satisfied with this health care gift.

jd_ 150416esd 2020-09-24 17:26
It's a very good model. It's much better than expected on the whole. It's very practical. I used it at home first. It's good. I can stew and boil it. The logistics is very fast. It will arrive the next day. The workmanship is also good and exquisite.

j***a 2020-11-11 09:19
Take it home and open it for use. It's really good. Many things can be stewed. For example, the lotus seed and red jujube soup I stewed is very nourishing. The size of the cup is also moderate, about 500ml, with two bowls. The whole health cup set looks very high-grade and I like it very much. Like friends can start.

jd189755svv 2020-09-22 11:32
Very well packed, very good style. I can recommend this one.

srkKqJoRkNgD 2020-11-21 15:14
The commodity quality is very good. It is very convenient to boil flower tea and occasionally stew porridge.

Ju * * * 2 2020-11-09 16:26
The health cup is very good. I like it very much. The cup has a good sense of design. It has powerful hot cooking function and is very practical.

j***c 2020-12-08 11:28
The cup style is pretty good. The heating effect of the coaster is very good. I'm generally very satisfied.

j***s 2020-12-15 15:29
Overall, the quality is very good. It's also very convenient for me to cook.

j***V 2020-11-30 14:58
The goods have been received. The packaging is exquisite, small and portable, and the electricity consumption is not large. You don't need to drink cold when drinking warm. It won't overflow and don't paste the bottom. It's really great.

melon 2020-12-30 16:59
In terms of compactness, it costs no electricity, has high appearance value and fast logistics. This health cup is very good, the cooking is also very good, the appearance is good, and it is very good to use.

jd_ 1861665941 2021-01-11 15:19
It's cold. A health cup is really practical and easy to use. It can cook a lot of things. It has an atmospheric appearance. It's green and inlaid with gold. It's very advanced. I like it very much.

jd_ 182505ykue 2021-01-13 16:34
It's also very convenient to cook pearl milk tea with it. Personally, I think it's great. The amount of cooking is also enough for one person, which is very practical and convenient.

jd_ 156437vil 2020-12-16 09:54
There is a gift box, the function is also very practical, and it is very convenient to cook. Cook corn ribs soup. It tastes good.

jd_ 387777230 2020-09-11 05:35
like it very much.

BLACK * * * 6 2020-10-20 00:40
It's very practical. It has a lot of functions. It's convenient to stew. It's very convenient to go out and use it inside. After reading the functions, I bought a dozen or so.

j***8 2020-5-26 08:55
I bought this kind of health preserving electric stew cup. The style is quite exquisite, and there are many practical functions. On the whole, it's still very good.

L7777799999 2020-12-26 10:51
Easy to use.

jd_ Pig's 2020-10-22 07:10
not bad. Just no gift bag.

ead 2021-01-24 13:53

It's very convenient to cook bird's nest. It feels very easy to use. A good gift.

super21jdmau 2020-09-13 17:01
Very good appearance. I like it very much. It's used to make tea.

Lllalalal 2021-02-09 23:27
The health cup is very suitable. The quality is very good. I feel very good.

8***3 2020-09-11 17:40
very nice. Think about it. It's very practical and cost-effective. It can be used to cook porridge.

Brother_1 2020-05-15 12:17
The color is super beautiful, easy to use, economical and affordable.

wsqdm123 2020-09-18 17:46
Very practical. I often cook snacks and health tea. The quality is very good.

oseyo 2020-12-26 10:52
The health cup is made very carefully.

jd_ 158013qhi 2020-12-26 09:05
The gift health cup is good. I really like it.

j***1 2021-01-28 09:48
Partiality.

Luka_ky 2020-07-14 12:22
Style: Great
Applicable crowd: good gift.

jd_ 180619ryh 2020-09-12 16:39
Very good.

---

3. Water cup, 3 negative reviews, with identity clues

jd_ 133585fdi 2021-02-24 09:55
The **women's Day** gift bought for the female employees at the bottom. The quality of the health cup is very good and the workmanship is exquisite. My **girlfriend** should like it very much.

PafatvpjpHBA 2020-10-30 11:57
It's very good. The workmanship is good. The order was placed last night and arrived in the morning. It was given to the **staff** on Thanksgiving. The style is very beautiful and practical. There are many main functions. I bought 50 sets and several boxes are very heavy. I believe everyone

will be satisfied with this health care gift.

jd_ 150416esd 2020-09-24 17:26
It's a very good one. The gifts given to **employee**s in the store during the Mid Autumn Festival are much better than expected. It's very practical. I used them at home first. It's good. I can stew and boil them. The logistics is very fast. They arrive the next day. The workmanship is also good and exquisite

j***a 2020-11-11 09:19
This is a gift for the annual meeting prepared by the **company**. I took it home and opened it for use. It's really good. Many things can be stewed. For example, the lotus seed and red jujube soup I stewed is very nourishing. The size of the cup is also moderate, about 500ml, with two bowls. The whole health cup set looks very high-grade and I like it very much. Like friends can start.

jd189755svv 2020-09-22 11:32
It's cheap. I don't like it very much. It's idle in the **dormitory**.

srkKqJoRkNgD 2020-11-21 15:14
I purchased 100 sets for the **company**. The quality of the goods is very good. Everyone in the office sends one set. Mine is very practical in the **office**. It is very convenient to cook flower tea and occasionally stew porridge.

Ju * * * 2 2020-11-09 16:26
The health cup is a very good gift for **employee**s. The end of the year gifts and year-end prizes are given to employees. **Employee**s like it very much. The cup has a good sense of design, powerful hot cooking function and is very practical.

j***c 2020-12-08 11:28
The cup style is pretty good, and the heating effect of the coaster is very good. This time, I bought two or three hundred gifts for the **employee**s and **leader**s of each branch for the third anniversary. I'm generally very satisfied.

j***s 2020-12-15 15:29
The company's annual meeting gifts that **my boss** asked me to buy are of good quality on the whole. It's also very convenient for me to cook. The staff liked it and the boss was very satisfied.

j***V 2020-11-30 14:58
It was originally a birthday gift for **my mother**, but the charging line of the product was too thin. I didn't dare to use it for the elderly. So bad.

Xiaosu_melon 2020-12-30 16:59
In terms of compactness, it doesn't cost electricity and has a high appearance value. It's very convenient to use it at **work in the company**. Logistics is also fast. This health cup is very good, the cooking is also very good, the appearance is good, and it is very good to use. Nice new year

gift.

jd_ 1861665941 2021-01-11 15:19
It's cold. A health cup is really practical. This is the gift I bought for my **girlfriend**. It's really easy to use. It can cook a lot of things. It has an atmospheric appearance. It's green and inlaid with gold. It's very high-grade. I like it very much.

jd_ 182505ykue 2021-01-13 16:34
It's also very convenient to cook pearl milk tea with it. Personally, I think it's great. The amount of cooking is also enough for one person, which is very practical and convenient. My **girlfriend** is the most satisfied Christmas present.

jd_ 156437vil 2020-12-16 09:54
The annual meeting accompanying gift given to **employee**s has a gift box, the function is also very practical, and it is very convenient to cook. Cook corn ribs soup. It tastes good.

jd_ 387777230 2020-09-11 05:35
I don't like it. After heating in the office all afternoon, the water is still warm. How to say, the temperature is a little low and of little use.

BLACK * * * 6 2020-10-20 00:40
It's very practical to give gifts to **customer**s and the **company**'s Mid Autumn Festival employees on the national day. It has many functions. It's convenient to stew. It's very convenient to go out and use inside. It's relatively good to give gifts. I've seen a lot of functions. I bought a dozen or so and plan to renew some.

j***8 2020-5-26 08:55
The gift of 520 for my wife happened to be our **wedding** anniversary, so I bought this kind of health preserving electric stew cup. The style is more exquisite and has more practical functions. On the whole, it's still very good.

claw 2020-12-26 10:51
In the **leader**'s office, the leader bought it, so he also bought one at home, which is easy to use

jd_ Pig's 2020-10-22 07:10
Teacher's Day gift for teachers, good. I just lose without a gift bag.

ead 2021-01-24 13:53
Women's Day gifts. Every female colleague has holiday gifts for female employees. They are very convenient for cooking bird's nests. It feels very easy to use. A good gift.

super21jdmau 2020-09-13 17:01
The Christmas gift bought in advance for the female colleague in pursuit. The **female colleague** liked it very much. She said that the appearance was very good. She liked it very much. She often

used it to make tea. I was very happy to see her use it.

Nnnnnn 2021-02-09 23:27
The **women**'s Day gift I bought for my mother, the health cup, is very suitable. The quality is very good. I feel very good. **My mother** likes it very much.

8***3 2020-09-11 17:40
Very nice. It's very practical and cost-effective. It's a birthday gift for **girlfriend**. He can use it to cook porridge.

Hhgmmm1 2020-05-15 12:17
Super beautiful color, especially suitable for **girl**s, easy to use, economical and affordable.

wsqdm123 2020-09-18 17:46
Especially **girl**s are very practical. I have one in my **office**. I often cook snacks and health tea. The quality is very good.

Rose 2020-12-26 10:52
It is very suitable for making gifts. This health cup is a holiday gift I intend to give to **my mother** before women's day. The workmanship of the health cup is very meticulous.

jd_ 158013qhi 2020-12-26 09:05
The prizes purchased at the **company**'s annual meeting are pretty good. There are a lot of employees. This time they need to be in a hurry, and the logistics is very fast. It was delivered as expected.The gift health cup is very good. I really like it.

j***1 2021-01-28 09:48
As a company gift, **colleagues** like it very much.

Luk_ Sky 2020-07-14 12:22
Style: Great.
Applicable crowd: it's good to send **teacher**s.

jd_ 180619ryh 2020-09-12 16:39
It was sent to the **leader**. The feedback was very good. Considerate service.

---

4. Water cup, 3 negative reviews, no identity clue

jd_ 133585fdi 2021-02-24 09:55
The health cup is of very good quality and exquisite workmanship. I like it very much.

PafatvpjpHBA 2020-10-30 11:57
Very good. The workmanship is good. The order was placed last night and arrived in the morning.

The style is very beautiful and practical. There are many main functions. I bought 50 sets and several boxes are very heavy. I believe everyone will be satisfied with this health care gift.

jd_ 150416esd 2020-09-24 17:26
It's a very good model. It's much better than expected on the whole. It's very practical. I used it at home first. It's good. I can stew and boil it. The logistics is very fast. It will arrive the next day. The workmanship is also good and exquisite.

j***a 2020-11-11 09:19
Take it home and open it for use. It's really good. Many things can be stewed. For example, the lotus seed and red jujube soup I stewed is very nourishing. The size of the cup is also moderate, about 500ml, with two bowls. The whole health cup set looks very high-grade and I like it very much. Like friends can start.

jd189755svv 2020-09-22 11:32
The packaging was not good and the product was delivered. The product was damaged and gave poor comments.

srkKqJoRkNgD 2020-11-21 15:14
The commodity quality is very good. It is very convenient to boil flower tea and occasionally stew porridge.

Ju * * * 2 2020-11-09 16:26
The health cup is very good. I like it very much. The cup has a good sense of design. It has powerful hot cooking function and is very practical.

j***c 2020-12-08 11:28
The heating effect of the coaster is average, easy to contact, poor, dissatisfied.

j***s 2020-12-15 15:29
Overall, the quality is very good. It's also very convenient for me to cook.

j***V 2020-11-30 14:58
The goods have been received. The packaging is exquisite, small and portable, and the electricity consumption is not large. You don't need to drink cold when drinking warm. It won't overflow and don't paste the bottom. It's really great.

melon 2020-12-30 16:59
In terms of compactness, it costs no electricity, has high appearance value and fast logistics. This health cup is very good, the cooking is also very good, the appearance is good, and it is very good to use.

jd_ 1861665941 2021-01-11 15:19
It's cold. A health cup is really practical and easy to use. It can cook a lot of things. It has an

atmospheric appearance. It's green and inlaid with gold. It's very advanced. I like it very much.

jd_ 182505ykue 2021-01-13 16:34
The amount of cooking is not enough for one person. It is not practical. Not recommended to buy.

jd_ 156437vil 2020-12-16 09:54
There is a gift box, the function is also very practical, and it is very convenient to cook. Cook corn ribs soup. It tastes good.

jd_ 387777230 2020-09-11 05:35
like it very much.

BLACK * * * 6 2020-10-20 00:40
It's very practical. It has a lot of functions. It's convenient to stew. It's very convenient to go out and use it inside. After reading the functions, I bought a dozen or so.

j***8 2020-5-26 08:55
I bought this kind of health preserving electric stew cup. The style is quite exquisite, and there are many practical functions. On the whole, it's still very good.

Claw 2020-12-26 10:51
Easy to use.

jd_ Pig's 2020-10-22 07:10
not bad Just no gift bag.

PencilHH 2021-01-24 13:53
It's very convenient to cook bird's nest. It feels very easy to use. A good gift.

super21jdmau 2020-09-13 17:01
Very good appearance. I like it very much. It's used to make tea.

2021-02-09 23:27
The health cup is very suitable. The quality is very good. I feel very good.

8***3 2020-09-11 17:40
very nice. Think about it. It's very practical and cost-effective. It can be used to cook porridge.

llllla 2020-05-15 12:17
The color is super beautiful, easy to use, economical and affordable.

wsqdm123 2020-09-18 17:46
Very practical. I often cook snacks and health tea. The quality is very good.

oseo 2020-12-26 10:52
The health cup is made very carefully.

jd_ 158013qhi 2020-12-26 09:05
The gift health cup is good. I really like it.

j***1 2021-01-28 09:48
Partiality.

luka_ Sky 2020-07-14 12:22
Style: Great.
Applicable crowd: good gift.

jd_ 180619ryh 2020-09-12 16:39
Very good. Considerate service.

Study 2

1.Portable juicer, positive reviews with identity cues

jd_ 133585fdi 2021-02-24 09:55
Considering **business trips**, you can drink fresh juice when working. Most of the traditional Juicers are large and choose the mini type. Brand and price are the factors to be considered. After all, it is economical to be diligent and thrifty. Finally, after self screening, Jiuyang portable Juicer was selected. This juicer is small in size, split design, easy to clean, and can meet my basic needs.

PafatvpjpHBA 2020-10-30 11:57
Bring two cups. After beating the juice, you can take it directly to the **company** to drink, a glass of juice every day. Nutritious, delicious and healthy.

jd_ 150416esd 2020-09-24 17:26
In order to lose weight, the mini juicer is small and convenient. It can be put in the bag every day. It can be used at the **company**'s home. It has a high white appearance and a quiet voice

j***a 2020-11-11 09:19
Every time an apple is just right, a pear is just right, and you can make juice quickly. I think it takes about 30 seconds. If you have a **tight working time** in the morning and just have a juice milkshake, just change the cover. It's really practical and convenient.

jd189755svv 2020-09-22 11:32
You can **start working** as soon as you press it. The operation is simple and convenient. The juice

is more delicate. It is used to make all kinds of vegetable mud and vegetable juice for your baby. It is super easy to use!

srkKqJoRkNgD 2020-11-21 15:14
It's really a mini juicer. I developed new functions and gave our dog fruit to drink. Hahaha, it's not noisy and juices quickly.You can make a glass of juice between **classes**.

Ju * * * 2 2020-11-09 16:26
It's important to make complementary food for **your baby**. It's clean and convenient, and the noise is not very big. Of course, in addition to complementary food, you can also freshly squeeze fruit juice, which is easy to clean. It's worth buying. You deserve it.

j***c 2020-12-08 11:28
It's very delicate, very good and feels very good. I recommended my friend to buy it. I think it's very good to buy it at this price. The sound is OK. It's not as quiet as expected, but the matching cup is very good. It's also a petty bourgeoisie. I think it's really cost-effective. If the **student** party and the **elderly**, I especially suggest staying.

j***s 2020-12-15 15:29
It's very small and mini. It's a small box. You can take it with you on business or put it in the **office**. The green one is very beautiful and suitable for summer.

j***V 2020-11-30 14:58
It's actually bad! It can't be repaired at all, rubbish!

melon 2020-12-30 16:59
Baby, I got it! It's very convenient to have a cup of slimming juice before **work** every morning. The cup is also very easy to wash. It's OK to flush the faucet. It's also easy to carry to five-star praise!

jd_ 1861665941 2021-01-11 15:19
Very good, very convenient! Successfully replace the heavy juice machine at home! It is easy to clean. Replace the bottle cap and flush the water with a knife. It's good to bring a bottle of juice when you **go out to work**.

jd_ 182505ykue 2021-01-13 16:34
It's very convenient and has a high appearance value. The fruit juice is very delicate and easy to clean. Ideal for office or short-term **business travel**.

jd_ 156437vil 2020-12-16 09:54
It's simple. After using it, I think the speed is OK and quite exquisite. In the afternoon, I'll buy fruit to try. I think it's very suitable for **student**s. It's more convenient. The price is beautiful and cheap.

jd_ 387777230 2020-09-11 05:35
I really like it. It's better than expected. It's also convenient for **children** to live in school. I will continue to buy it back.

BLACK * * * 6 2020-10-20 00:40
The juicing effect is particularly good. It takes only ten seconds to squeeze a cup of fresh fruit juice. It's very convenient to drink it at **work** during the day! Highly recommended! Pink strawberry yogurt, red tomato juice!

j***8 2020-5-26 08:55
The goods have been received. I made milkshakes several times. I feel that they taste very good. It's also very simple to copy. Just press them down for a few seconds, then remove the drill bit, screw the cup cover, and take them to **work**. It's great to replenish energy.

claw 2020-12-26 10:51
I don't like it. It's not easy to use at all.

jd_ Pig's 2020-10-22 07:10
It's not used yet. I don't know how long it can be used. I hope it can be used longer. I bought it to make a milkshake. It's estimated to score twice, because it's really small and mini. The sister of the **student union** should buy one.

ilhead 2021-01-24 13:53
In fact, it's the second time to buy the same white. There's a special price this time. Take two cups and try it. It's very fast. Squeeze the juice quickly. The cup has no peculiar smell and juices smoothly. It is very suitable for **office worker**s to bring breakfast in the morning. It is convenient, fast and nutritious.

super21jdmau 2020-09-13 17:01
I tried to squeeze orange juice when I bought it. **My family** like the taste and the power is very strong! Moreover, the squeezed juice is very delicate and easy to rinse after juicing.

Hhhh 2021-02-09 23:27
Very good. **My daughter** has found a new thing. The juice is very thin. The orange juice can see the broken skin of the orange pulp, but there is no pulp in apples, pears, bananas, peeled kiwi fruit and so on, which are paste or semi liquid.

8***3 2020-09-11 17:40
Jiuyang's small electrical appliances are still quite good. It's light and convenient to buy juice for your **girlfriend**. It's great!!! recommend!

Brother_1 2020-05-15 12:17
Don't buy it. If you buy it, you'll be fooled. It won't work at all. A rotten product can't even hit an apple.

wsqdm123 2020-09-18 17:46

After receiving the milk shake, give the **child** a drink. The operation is simple. The cup is small and exquisite. The child can operate it by himself. The sound is within the acceptable range.

Rose_yo 2020-12-26 10:52

This is very small and does not occupy an area! When making juice, put the fruit into a portable cup and add a little water or milk. I'm used to putting some honey, change the cover of the blade, and then install it on the host. Press and hold the switch button for a few seconds, and a cup of juice rich in fruit fiber will be OK! It's more convenient to take it down after typing, change the cup cover, and directly put it in the bag and take it to the **company** for a drink!

jd_ 158013qhi 2020-12-26 09:05

It's fast to make juice. You can get a cup in a minute or two. It's necessary for **home,** travel and **work**. Juicing things are better than Jiuyang.

j***1 2021-01-28 09:48

The commemorative prizes purchased for the **company**'s year-end activities are also very beautiful when they are purchased in time for the activities. After opening them, they are small and exquisite. They are suitable for daily singles or for use in the unit. The prices are good. What's recommended.

Luka_sky 2020-07-14 12:22

It's small and easy to operate. It's very convenient for **office workers**. Whether operation or cleaning!! A full bottle of Pitaya doesn't add water, and a thick juice is fast. It's delicious!

jd_ 180619ryh 2020-09-12 16:39

The order was placed last night and arrived this morning. Don't be too fast! In fact, it's the second time to buy the same white. There's a special price this time. Take two cups and try it. It's very fast. Squeeze the juice quickly. The cup has no peculiar smell and juices smoothly. It is very suitable for **office workers** to bring breakfast in the morning. It is convenient, fast and nutritious.

2.Portable juicer, negative reviews with identity cues

jd_ 133585fdi 2021-02-24 09:55

Most of the traditional Juicers are large, so they choose the mini type, and the brand and price are the factors to be considered. After all, only when they are economical can they be diligent and thrifty. Finally, after self screening, Jiuyang portable Juicer was selected. This juicer is small in size, split design, easy to clean, and can meet my basic needs.

PafatvpjpHBA 2020-10-30 11:57

Bring two cups. A glass of juice every day. Nutritious, delicious and healthy.

jd_ 150416esd 2020-09-24 17:26
In order to lose weight, the mini juicer is prepared. Because it is small and convenient, the white color value is very high, and the sound is not noisy.

j***a 2020-11-11 09:19
Every time an apple is just right, a pear is just right, and you can make juice quickly. I think it takes about 30 seconds. It's just a cup of juice milkshake. Just change the cover. It's really practical and convenient

jd189755svv 2020-09-22 11:32
You can start working as soon as you press it. The operation is simple and convenient. The juice is more delicate. It's super easy to use!

srkKqJoRkNgD 2020-11-21 15:14
What a mini juicer. It juices quickly.

Ju * * * 2 2020-11-09 16:26
Clean and convenient is very important, and the noise is not very big. Of course, in addition to supplementary foods, you can also freshly squeeze fruit juice, which is easy to clean. It's worth buying. You deserve it.

j***c 2020-12-08 11:28
It's very delicate, very good and feels very good. I recommended my friend to buy it. I think it's very good to buy it at this price. The sound is OK. It's not as quiet as expected, but the matching cup is very good. I think it's really cost-effective.

j***s 2020-12-15 15:29
It's very small and mini. Received the box. It's so small that it doesn't take up space. The green one is very beautiful and suitable for summer.

j***V 2020-11-30 14:58
I was going to buy it for **school**. I was drunk and tried it today. It was actually bad! It can't be repaired at all, rubbish!

melon 2020-12-30 16:59
Baby, I got it! It's very convenient to have a cup of slimming juice. The cup is also very easy to wash. Just flush the faucet. It's also easy to carry to five-star praise!

jd_ 1861665941 2021-01-11 15:19
Very good, very convenient! Successfully replace the heavy juice machine! It is easy to clean. Replace the bottle cap and flush the water with a knife.

jd_ 182505ykue 2021-01-13 16:34
It's very convenient and has a high appearance value. The fruit juice is very delicate and easy to

clean.

jd_ 156437vil 2020-12-16 09:54
It's simple. After using it, I feel that the speed is OK and quite exquisite. In the afternoon, I'll buy fruit to try. It's much more convenient. The price is good and cheap.

jd_ 387777230 2020-09-11 05:35
I love it. It's better than I thought.

BLACK * * * 6 2020-10-20 00:40
The juicing effect is particularly good. It takes only ten seconds to squeeze a cup of fresh fruit juice. It is highly recommended! Pink strawberry yogurt, red tomato juice!

j***8 2020-5-26 08:55
The goods have been received. I have made milkshakes several times. I feel they taste very good. It is also very simple to copy. Just press them down for a few seconds, then remove the drill bit and screw the cup cover

Claw 2020-12-26 10:51
In the afternoon, I had two cups of milk (pitaya, milk orange) in the **student dormitory**. The residue could not be washed clean and was not easy to use.

jd_ ig's 2020-10-22 07:10
It's not used yet. I don't know how long it will last. I hope it will last longer. I bought it to make a milkshake. It's estimated to score twice, because it's really small and mini.

Penci1 2021-01-24 13:53
In fact, it's the second time to buy the same white. There's a special price this time. Take two cups and try it. It's very fast. Squeeze the juice quickly. The cup has no peculiar smell, the juice is smooth, convenient, fast and nutritious.

super21jdmau 2020-09-13 17:01
I tried to squeeze orange juice when I bought it. I like the taste and strong power! Moreover, the squeezed juice is very delicate and easy to rinse after juicing.

Hhhh 2021-02-09 23:27
Very good. The juice is very fine. You can see the broken skin of orange pulp in orange juice, but you can't see any pulp in apple, pear, banana, peeled kiwi fruit and so on. They are all paste or semi liquid.

8***3 2020-09-11 17:40
Jiuyang's small electrical appliances are quite good. It's light and convenient to squeeze juice. It's great!!! recommend!

brother1 2020-05-15 12:17
Don't buy it. If you buy it, you'll be fooled. It won't work at all. There's a rotten product. You can't even hit the apple. I am going to return it **downstairs**. I'm going to be late for **class**.

wsqdm123 2020-09-18 17:46
After receiving the milk shake, I drank it. The operation is simple. The cup body is small and exquisite. I can operate it myself. The sound is within the acceptable range

Rose4 2020-12-26 10:52
This is very small and does not occupy an area! When making juice, put the fruit into a portable cup and add a little water or milk. I'm used to putting some honey, change the cover of the blade, and then install it on the host. Press and hold the switch button for a few seconds, and a cup of juice rich in fruit fiber will be OK!

jd_ 158013qhi 2020-12-26 09:05
It's fast to make juice. You can finish a cup in a minute or two. It's better to extract juice from Jiuyang.

j***1 2021-01-28 09:48
The price is very beautiful. After opening it, it's small and exquisite. It's suitable for daily use. The appearance and price are good. What's recommended.

luka_Sky 2020-07-14 12:22
Very compact, easy to operate, whether it is operation or cleaning!! A full bottle of Pitaya doesn't add water, and a thick juice is fast. It's delicious!

jd_ 180619ryh 2020-09-12 16:39
The order was placed last night and arrived this morning. Don't be too fast! In fact, it's the second time to buy the same white with two cups. Try it. It's very fast. Squeeze the juice quickly. The cup has no peculiar smell, the juice is smooth, convenient, fast and nutritious.

Study 3

1.Neck massage machine, aspirational groups

The positive rate of the product is 98%. The following are typical reviews:

s***n 2020-07-27 11:19
This is really comfortable! It is especially suitable for **desk workers**, cervical pain and discomfort. Press it to relieve too much, and get immediate results! And it's light and easy to carry. All kinds

of bags go as soon as they are loaded. It takes n long to charge once. That's great.

g***4 2020-06-23 09:18
Wall crack with bad cervical spine is recommended ~! During this period of time, the work was busy, which led to the beginning of cervical spondylosis that had not been committed for many years. Therefore, the new model bought has the function of hot compress. Hot compress combined with physiotherapy can alleviate a lot in about five minutes. It's really great! Bow head family must prepare one. You can take it with you during **long-distance travel** and **driving**. USB charging is also very convenient.

l***v 2020-05-14 23:03
The whole is compact and easy to carry. The remote control is very convenient to use. The remote control can be sucked on the side of the machine and is easy to find. There is no need to turn off manually after massage. It is very humanized design. The massage strength is highly graded. Different strength is selected according to different choices. There are three modes. **Facing the computer every day**, my neck is very hard and painful. I massage twice a day. There is no joint sound when my neck moves. I am very satisfied with the shopping experience!

Snow * * * V 2020-06-26 12:35
"It can be used in all kinds of occasions, such as **office**, rest, family, **driving**, etc
It's mainly in the car. It's very convenient to bring a remote control. The commute is 10 minutes. Why not enjoy it while driving.

w***6 2020-11-25 14:13
I **work in the hospital**. I usually write medical records every day for a long time. Neck pain has been regarded as an occupational disease for several years. This time, this massager has fine workmanship first. If the color can be a little more, all kinds of modes have been tried. I especially like to open hot compress. After applying the neck, I don't feel so sore. This massager can bring its own voice broadcast. It's very convenient and easier to use than the remote control More, if the battery capacity is bigger, it will be perfect.

j***u 2020-12-02 17:12
I like it very much. It's easy to use. I've been **sitting in the office** for a long time, using computers and playing mobile phones. Over time, I have cervical spondylosis and my neck hurts very much. Since I bought this, there are not only four modes, but also fever. I feel very comfortable every time I use it. I can adjust the grade size according to my own feeling. If it's too large, there will be a sense of acupuncture, so it's good to adjust it until I feel comfortable! Highly recommended! Great!

jdpinnki 2020-02-21 10:12
Those who play with **computers or mobile phones for a long time can enter one**. They can massage at any time and carry it conveniently. When they first press it, they feel a little pricked. They can choose hot compress together to alleviate the pain of pricking a little. However, after pressing it, they feel very comfortable and relaxed. In particular, hot compress feels super good. It

is recommended to many friends around them. It's really good!

Jane * * * 9 2020-07-07 15:24
People who have been **sitting in the office** for a long time have problems with their cervical vertebrae. This massage instrument is really good. I like its heating function best. When winter comes, the channels of the cervical vertebrae are blocked and easy to cold. This massage instrument can be worn all the time, and it is particularly comfortable to heat. The massage gear can be adjusted by ten levels, which can really alleviate cervical fatigue.

j***a 2020-06-22 09:14
I placed the order last night and received it today. The logistics is very good. Besides, this thing, champagne gold, is very advanced and beautiful. There are four modes to choose from. It's crisp and numb after wearing it. I tried it soon after I got it. The hot compress speed is very fast, and it's crisp and numb and hot. It's a sharp weapon to save **white-collar workers**!

j***1 2020-12-12 21:14
Highly recommended. It's really easy to use, very light and easy to carry. There are four modes to switch freely, and the heating function is very comfortable. You can enjoy it **in the office**, at home and **on the road**. This product is worth recommending.

Marizon_1 2020-09-15 12:14
The color is very good-looking, small and exquisite, easy to carry. After trying it for a period of time, I feel the effect is quite good. It can be used at home and **in the office**. The temperature and battery sensing can be adjusted in gear. In addition, there are voice prompts, which are very convenient and can effectively relax the neck. The pain has been relieved a lot.

V***g 2020-02-06 13:28
Gifts bought for **leaders**, I haven't used them, and I don't know the specific effect. They should be high-end!

j***b 2020-07-11 19:22
Very good goods, a gift for **teachers** on teacher's day, very suitable! Bluetooth, easy to operate! There are many gears, which is suitable for relaxing your neck when you sit **in the office** for a long time! It is very comfortable to use and can relieve the fatigue of the neck!

Yu * * * Xi 2021-01-12 17:20
Now I **sit in the office** facing the computer every day. When I get home, my waist and neck are sour. This massage instrument can just **relax after work** and relieve the fatigue of the day. Multiple modes, can be heated, and large screen display, easy to operate, very good.

l***3 2021-02-19 08:28
I think this massage instrument is very good. It has the function of arrangement and combination of heating and strength, and can be applied to many people. However, I generally use only 5-7 strength. Massage is a bit like current. After use, the cervical spine has a aftertaste. It is very

suitable for **urban white-collar workers**. It looks big, but it is actually small and light.

***YUAN 2020-06-17 09:59
I've been thinking about it for a long time. I finally did the activity. I placed an order in the morning and sent it in the evening. I quickly charged it and tried it. The electric impulse is very powerful and crisp, but I feel stiff and relaxed. Long term use can alleviate the sore neck. I like it very much. Moreover, it is small and exquisite in shape and light in weight. It can stand by for 8 days after charging. **Office white-collar worker**s often use computers. It is very effective to alleviate cervical problems. It is recommended that you prepare one. Health is the most important.

Xing * * * Italy 2020-11-13 22:58
Cervical spondylosis has been harassed for many years. Acupuncture, massage and needle knife treatment have been tried. As a daily estimate of **office hours**, it is still very moving and can not replace manual massage. The shape is fashionable and can be used frequently **in the office**. It should have alleviating effect.

z***6 2021-01-12 22:01
I always feel uncomfortable in my neck and cervical spine **when I work at my desk for a long time**. I searched the massage instrument online and recommended this one. I never thought I could buy such a good baby at such an affordable price,? Very cost-effective. After using it for a while, the cervical pain has been greatly improved. I like it very much.

j***0 2020-10-09 23:05
This product is an electric stretching massage, which has achieved the ideal effect. The workmanship is very exquisite. The neck will be much more comfortable after massaging for a while. It is necessary to start often **in the office**. It is worth it!

j***d 2020-05-09 13:25
It's a very easy-to-use neck massage instrument. I tried it immediately after receiving the goods. It's very good to click it. It's suitable for people **in sedentary offices**. The Bluetooth applet is also very convenient and very comfortable to use.

MIAO_ 1 2020-05-27 18:40
Massage is very effective and will feel much easier. Usually, I always use computers and mobile phones too much, and my neck and shoulders ache. This has a heating function, especially when I'm on a **business trip**. It can also be used on the plane, which can effectively alleviate the pain. Good!

LolleJO 2020-08-17 23:04
After receiving the goods, I experienced it. The massage is very comfortable, with a remote control, easy to operate, fashionable and good. There are three modes of massage, with 15 gears, heating function and fast charging speed. It is very suitable for people **working on computers** in the office all day. The price is relatively affordable.

m***0 2020-09-22 10:38

I tried the same product of **my colleagues** and immediately placed an order to buy it, one in the office and the other at home. It's really comfortable, especially when the electronic pulse is pricked like a small needle. It's crisp and good. Very suitable for relaxation after work.

LLtt 2020-08-17 11:09

Nowadays, the cervical pain of occupational diseases is terrible. It can be relieved. It's OK. It may be a little uncomfortable after a long time. It's easy to carry. It's a sharp weapon **at work**. Buy it!

World insect123 2020-07-23 08:22

I bought it after **my colleagues** introduced it. It's light and comfortable. It's only the size of a palm. The operation is also very simple. There are four modes to operate. I bought one for myself and my husband respectively, which is suitable for **office workers**.

j***1 2020-10-19 16:29

It's easy to use. It's very comfortable to massage before going to bed every day. It's a very good product. The massage strength can be adjusted at any time according to needs. It's very suitable for **office worker**s facing the computer every day. It saves the time spent on the road when they go to the store for massage. You can massage while **playing with children**, which can alleviate fatigue without delaying **parent-child time**.

j***w 2021-02-24 17:58

The personal experience is very good.It is small and convenient, heating speed is very fast, and there are a variety of modes to choose, which is suitable for the needs of different people. It is an indispensable tool for the treatment of cervical vertebra **in the office**.

j***o 2020-12-19 14:50

I can't stand facing the computer all day **at work**. I heard it's easy to use. I bought it and thought it would have adverse reactions. Who knows it's very comfortable. It's very suitable for us who sit **in the office** every day to protect our cervical spine.

Xing * * * Guo 2020-02-25 10:32

The occupational disease **in the office** can not be avoided is the cervical spine. No matter how much attention is paid, it is always inevitable that the cervical spine is sore. I don't have time at ordinary times. **I can't go to the hospital for professional treatment**. I've been looking for alternatives. This therapeutic instrument is easy to carry and has a strong massage feeling. It's very good. It can be treated at any time **in the office**. Recommend.

Latticeee 2020-08-19 18:05

Neck massage is fast and comfortable. The gear can be adjusted. You can wear it while **working** during the day. It's very convenient.

2.Neck massage machine, in-groups

The positive rate of the product is 98%. The following are typical comments:

s***n 2020-07-27 11:19
This massage instrument is good as a whole. It's beautiful and fashionable. It's a birthday gift for your **roommate**.

g***4 2020-06-23 09:18
The massager is small, exquisite and beautiful. Fully charged can be used for 5 or 6 times. Not heavy, very light. Put it in your **schoolbag**. You can take it to class or between **class**es.

l***v 2020-05-14 23:03
It's very good to use. Massage with different frequencies has different feelings. It's also very convenient to carry. It's especially cost-effective to buy on **dormitory** and use it together.

Snow * * * V 2020-06-26 12:35
It's very comfortable to take. The heating massage is very good. It's fashionable and fashionable. Cervical vertebra is very uncomfortable when you go back to the dormitory after **a day's class**, because you bow your head for a long time. With a massage instrument, you can massage at any time.

w***6 2020-11-25 14:13
Students can afford it. Mobile phone control is also very convenient, with almost no sound, which is very suitable for one's leisure.

j***u 2020-12-02 17:12
Students are now **taking online classes**. They can't stand using the computer for too long. This equipment is still good, the strength is just right, and it can be heated. It feels good.

jdpinnki 2020-02-21 10:12
My daughter feels practical. She studies **homework** every day. Her neck and cervical spine are very uncomfortable. She uses it several times a day. It's convenient to use it while learning for the month.

Jane * * * 9 2020-07-07 15:24
The highest gear is 14. Drive to the maximum gear, drive to the edge of the city, roll down the window and let the whole person fly. I'm no longer afraid of cervical pain. Oh, the welfare of the **student**.

j***a 2020-06-22 09:14
As a **child**, i am tired of **studying** every day. The operation is simple and convenient.

j***1 2020-12-12 21:14

The online review is very good. An online red product has a good appearance and feel of use. It has a great sense of texture and technology. **Children** who often learn can try it. It's great on the whole and highly praised!

Lisong 111 2020-09-15 12:14
As a **college** student who has been studying in front of a computer for a long time, this really improves the happiness of life. I like the new color very much. There are two temperature levels and four modes. I prefer intelligent mode and physiotherapy mode for the time being. Now I use it once in the morning and once in the evening. It's very comfortable!

V***g 2020-02-06 13:28
It's really valuable. The price is very cheap. Massaging for 15 minutes a day is very good for the cervical spine. It's very suitable for the **student**. I feel that hot compress is really comfortable.

j***b 2020-07-11 19:22
Very easy to use, there are many gears, which is very suitable for **student**s and office workers. N ice!

Yu * * * Xi 10:21, November 29, 2019
Beautiful appearance, simple keys, very comfortable massage, approximate touch is stimulated by current, there are several different massage modes, simple operation, suitable for **dormitory** use!

l***3 2021-02-19 08:28
A very good cervical massager, with exquisite packaging, fine workmanship and small size, is easy to carry out. Take it out and try it. The massage effect is particularly good. You feel tired after **postgraduate entrance examination**. Put cervical massage. First wet the electric shock and cervical spine with water, and then start regular massage. After massage, you feel very comfortable and remove fatigue. It's a good massager. It's worth buying.

sport11 2020-06-17 09:59
The massager is very comfortable to use and relieves neck fatigue. It can be used while **learning** to save time.

Xing * * * Italy 2020-11-13 22:58
It's easy to use. The effect of physical therapy is good. The key is to stick to it! Introduced to the **students** and said it was very comfortable. I liked it.

z***6 2021-01-12 22:01
We buy 3 sets in our **dormitory**. Cervical spine problems are common. I feel that the power consumption is still very large. The pulse force adjustment function is good, and the heating function is the most comfortable. People who are tired of **learning** every day need it.

j***0 2020-10-09 23:05
It's a very suitable birthday gift for my **classmate**s. I specially asked her to try it and tell me her

feelings. She may be more tolerant and doesn't feel strong. I always think this one has high cost performance and high appearance value. I'm going to buy it myself.

j***d 2020-05-09 13:25
It took two days. It's super comfortable. In order to take the **graduate entrance examination**, I bow my head every day. It feels good to use it.

miao1 2020-05-27 18:40
Relieve cervical pain! **Student**s use it well!

Lollejo 2020-08-17 23:04
Firstly, the workmanship of the product is very exquisite, and the feedback strength of the keys is just good. Secondly, the effect is also as expected, which is basically consistent with the physiotherapy principle of the hospital. After use, it feels that it has a certain regulating effect. Finally, the power is relatively long-lasting, and the **children** can be brought to school seven times a week!

m***0 2020-09-22 10:38
Things are very good. Massage for 15 minutes each time. There are many gears and modes. It feels very comfortable after massage. It's suitable for **student**s to use ha!

llltt 2020-08-17 11:09
That machine is for children. I'm **tired from school** every day. I give him a massage while doing his **homework**.

111***123 2020-07-23 08:22
It's very comfortable to use in the **bedroom**. I feel that the pressure of **learning** all day has been released.

j***1 2020-10-19 16:29
There is a slight sense of acupuncture and a little pain. It feels much better if the gear is smaller. You can also **take it in class**. It is very convenient, easy to operate, and has a good effect.

j***w 2021-02-24 17:58
A birthday present for a friend. Students are very tired after **graduate school**. They can massage the cervical spine. Feedback is very good.

j***o 2020-12-19 14:50
Small things are of great use. The massage effect is very good. It can be used for **learning**. Massage for 15 minutes to eliminate fatigue and relax.

Xing * * * Guo 2020-02-25 10:32
Very good. It works well. It's very fashionable. It's OK to wear it **at school**. Good looking.

Lattice888ee　　2020-08-19 18:05
My brother often lowers his head when he **studie**s, so he bought it for him. He said it was good to use.

3.Neck massage machine, dissociative groups

The positive rate of the product is 98%. The following are typical comments:

s***n　　2020-07-27 11:19
Things are very good. I bought one for my **mother** and **mother-in-law** after the festival. It is said that it is very good to use. They are very satisfied. They all say it is very comfortable. The heating function is particularly practical and the massage is particularly comfortable. Unlike the traditional massager, it will give people an uncomfortable feeling for a long time. This can be adjusted by themselves. Good.

g***4　　2020-06-23 09:18
My **mother** always suffers from cervical vertebrae. I bought one and tried many models. Finally, I bought this one. There are more physiotherapy modes and low-grade hot compress than other styles. I used it as soon as I got it. I wasn't used to it at first. The electricity hurts very much. I got used to it a lot after using it several times. My neck is very comfortable and relaxed during massage. I'll have a look at it for a while, Good effect.

l***v　　2020-05-14 23:03
The new technology bought for my mother-in-law on **Mother's day**. The **old man** likes and is willing to accept new things. He sent two sets of crystal stickers with electrodes. The operation is simple and easy to use. There are three modes and 15 forces. I use force 7. My mother-in-law likes the feeling of strong current stimulation.

Snow * * * V 2020-06-26 12:35
It feels great to use. The voice prompt is clear and loud. It is suitable for the **elderly**.

w***6　　2020-11-25 14:13
It's very good. I bought it for my **parents' birthday**. The appearance and style are very good!
When used, there are many modes that can be adjusted. The vibration feels very good, and it's quite convenient to adjust.

j***u　　2020-12-02 17:12
Easy to use. It can increase and weaken according to your own feeling. The vitality mode, indirect electrotherapy, numb and soothing mode are push and play. It is recommended to use the strong mode for serious cervical vertebrae. It has the function of adjuvant therapy and voice prompt. The operation is simple and suitable for the **elderly**.

jdpinnki　　2020-02-21 10:12

The neck massage instrument bought for **my parent**s feels good after my father uses it! I hope it can help my parents' health a little! The product is very textured! The packaging is very exquisite! It's also very convenient to use! It's really good.

Jane * * * 9 2020-07-07 15:24
I think it's really good. **Parents** like it very much. Four gears are adjustable, skin friendly and comfortable, and relax the cervical spine. The temperature is also comfortable, warm but not dry, penetrating and soothing. Easy to use, very portable, often used when watching TV. When parents are **old**, it's a good choice to give this gift.

j***a 2020-06-22 09:14
The operation is simple and convenient. I feel comfortable after use. My **parent**s like it very much. Worth buying

j***1 2020-12-12 21:14
Recommended for **parents**.

Song 111 September 15, 2020 12:14
I bought it for the second time. My **mother** said it was good to use the birthday gift originally bought for my father, so I let them one by one, which is convenient to use. Baby received it. The quality is very good. The packaging is exquisite and the material is excellent. It's better than expected. It's very suitable to give gifts for your own use.

V***g 2020-02-06 13:28
It was delivered on the day of **March 8th**. It was bought as a gift for my **mother**. It is well packaged, compact and easy to carry.

j***b 2020-07-11 19:22
Because my **mother-in-law** has cervical spondylosis, it's a gift for mother's day. After a period of time, I felt good and bought one for my mother. Easy to operate, soft material. The heating function is very comfortable, and the gear can be adjusted by yourself. Mother watches TV, watches mobile phones, cooks and wears them every day.

Yu * * * Xi 2020-07-21 9:09
I bought it for the **old** man. It has been used for some time. The old man said that it takes about a quarter of an hour to do it twice a day. The cervical vertebra is comfortable and a lot of money. I hope the effect will be better and better. I feel that the details of the product are handled very well. The internal parts close to the skin are very soft and close to the skin. The operation is very simple. The old man will use it after trying it several times.

l***3 2021-02-19 08:28
I am **50 years old**. My cervical spine is bad. My colleagues recommend buying this one to relieve fatigue. It's really good to try it. Before it's useless, my neck is always sour and hard. It will relieve a lot after 15 minutes of massage. In addition, there are three modes, which are very comfortable.

hhhh1 2020-06-17 09:59
It's very convenient to buy a gift for the **elder**s, because the elders have prominent cervical vertebrae, which is convenient for massage and relieve the pain. Their parents like it very much. They experience it first when they get it. It's very comfortable

Xing * * * Italy 2020-11-13 22:58
I bought it for my **mother**. If your cervical vertebra is uncomfortable, buy it and try it.

z***6 2021-01-12 22:01
It's very easy for the **elderly** to use. It can be used every day. It's very lightweight, simple and fashionable. It has a high appearance value. There are many gears that can be adjusted.

j***0 2020-10-09 23:05
The **elderly** cervical spine is not very comfortable. I feel very good after reading the previous online recommendation. After I bought it, it's very comfortable to use it. It's better than I thought before. In particular, this large screen display is more convenient. The elderly are not very good at using mobile phones and can operate directly.

j***d 2020-05-09 13:25
My mother felt very comfortable. The quality and appearance were very good. It had a high sense.

Miao_111 2021-02-01 17:10
Cervical massage instrument is really good. **My father** uses it every day. There are several gears. It can also be heated according to his stress. It is very convenient to use. It can be used many times to charge electric energy. I'm tired of working all day. It's very comfortable! Worth having!

Lolleyo 2020-08-17 23:04
It's really suitable for dad, mom, Grandpa and grandma. I tried to massage the neck. It's really comfortable. I suggest that there are **old people at home**. It's really suitable for them to improve their cervical problems. A little love will really move them for a long time, and it's also good for their health. You can try it.

m***0 2020-09-22 10:38
A good massage instrument recommended by a friend. It's bought for **my parents**. My parents often feel uncomfortable when they are old. This one is very convenient to use. It doesn't delay watching TV and brushing their mobile phone. It's easy to liberate their hands. My parents can do it by reading the instructions.

kkktt 2020-08-17 11:09
I bought it for **my mother**. Because she always likes to find a tablet computer, I gave her one. It's very convenient to use.

1ii123 2020-07-23 08:22
The quality is good and the packaging is exquisite. It's really good to give to the e**lderly**. My mother likes it very much.

j***1 2020-10-19 16:29
It's very good for the **elder**s. It's simple to operate and easy to use. It's very comfortable to use.

j***w 2021-02-24 17:58
My mother and mother-in-law are uncomfortable with their cervical vertebra when they are old. It's small and exquisite.

j***o 2020-12-19 14:50
Suitable for the **elderly**, very good.

Xing * * * Guo 2020-02-25 10:32
It's for **Dad and mom**. Very useful.

Latticeee 2020-08-19 18:05
I bought it for **my mother**. She cervical vertebra instrument is small, and the effect is very good.

4.Neck massage machine, no identity clue

The positive rate of the product is 98%. The following are typical comments:

s***n 2020-07-27 11:19
Unlike the traditional massager, it will give people an uncomfortable feeling for a long time. This can be adjusted by yourself.

g***4 2020-06-23 09:18
There are more physiotherapy modes and low-grade hot compress than other styles. I used it as soon as I got it. I got used to it a lot after using it several times. The neck is very comfortable and relaxed when I press it. I'll have a look for a period of time and buy another one with good results.

l***v 2020-05-14 23:03
The operation is simple and easy to use. There are 3 modes and 15 forces. I use force 7. I feel strong current stimulation twice a day.

Snow * * * V 2020-06-26 12:35
The voice prompt is clear and the voice is loud.

w***6 2020-11-25 14:13
The things are very good. The appearance and style are very good! When used, there are many

modes that can be adjusted. The vibration feels very good, and it's quite convenient to adjust.

j***u 2020-12-02 17:12
Easy to use, the strong mode is very comfortable, and the push and play and electrotherapy are very comfortable. The intelligent mode is relatively gentle and the gear is adjustable. It can increase and weaken according to your own feeling. It has the function of adjuvant treatment, voice prompt and simple operation

jdpinnki 2020-02-21 10:12
The product is very textured! It's also very convenient to use! It's really good.

Jane * * * 9 2020-07-07 15:24
It's really good. I like it very much. Four gears are adjustable, skin friendly and comfortable, relaxing the cervical spine. The temperature is also comfortable, warm but not dry, penetrating and soothing. It's easy to use, very portable, and often used when watching TV.

j***a 2020-06-22 09:14
The operation is simple and convenient. I still feel comfortable after use. It's worth buying.

j***1 2020-12-12 21:14
It's really good to recommend it to you.

Song _5552020-09-15 12:14
It's easy to use. The quality is very good, the packaging is exquisite, and the material is excellent. It's better than expected. It's very suitable to give gifts for your own use.

V***g 2020-02-06 13:28
It was bought as a gift for yourself. It is well packaged, compact and easy to carry. The remote control is very convenient to use.

j***b 2020-07-11 19:22
I feel pretty good after using it for a period of time. It's easy to operate and soft. It's very comfortable with the heating function. The gear can be adjusted by myself. I wear it every day when watching TV, watching mobile phones and cooking.

Yu * * * Xi 2020-09-06 19:20
It's been used for some time. It's done twice a day for about a quarter of an hour. The cervical spine is comfortable and a lot of money. I hope the effect can be better and better. I feel that the details of the product are handled very well. The internal parts close to the skin are very soft and close to the skin.

l***3 2021-02-19 08:28
It will relieve a lot after 15 minutes of massage. In addition, there are three modes, which are very comfortable.

sport_1 2020-06-17 09:59
It's very convenient to massage and relieve the pain. I like it very much. I experienced it first when I got it.

Xing * * * Italy 2020-11-13 22:58
As a birthday gift, the effect is better than expected.

z***6 2021-01-12 22:01
It's very good and easy to use. It can be used every day. Very light, simple and fashionable.There are many gears that can be adjusted.

j***0 2020-10-09 23:05
I feel very good about this recommendation on the Internet. After I bought it, it's very comfortable to use it, which is better than I thought before. Especially this large screen display is more convenient and can be operated directly.

j***d 2020-05-09 13:25
I think it's very comfortable. The quality and appearance are very good. It feels advanced.

Xiaomiao_11 2020-05-27 18:40
The cervical massage instrument is really good. It can be heated according to your stress condition, and is very convenient to use. It can be used to recharge electric energy for many times. It can help you relax and lean quietly at night. It's very comfortable!

Lolleyo 2020-08-17 23:04
I tried to massage the neck. It's really comfortable. It can improve cervical problems and is also good for health. You can try it.

m***0 2020-09-22 10:38
Recommended by a friend, it's a very easy-to-use massage instrument. It doesn't delay watching TV and brushing your mobile phone. The operation is very simple, the charging model is relatively convenient, the strength of several gears is very good, and the appearance is also beautiful.

xiaott 2020-08-17 11:09
This is very convenient to use because it has voice prompt function, which is very practical!

chong123 2020-07-23 08:22
Good quality, exquisite packaging, really good, I like it very much.

j***1 2020-10-19 16:29
The things are very good, simple to operate, easy to use, very comfortable to use, and the neck is not so stiff.

j***w   2021-02-24 17:58
I like it very much. It's so small and exquisite.

j***o   2020-12-19 14:50
Suitable for use, very good.

Xing * * * Guo 2020-02-25 10:32
Bought for health preservation, highly praised.

Latticeee   2020-08-19 18:05
The cervical vertebra instrument is small and effective.
